# Hypermethylation-Associated Silencing of miR-125a and miR-125b: A Potential Marker in Colorectal Cancer

**DOI:** 10.1155/2015/345080

**Published:** 2015-11-26

**Authors:** Hui Chen, Zhiying Xu

**Affiliations:** ^1^Department of Gastroenterology, People's Hospital of Taizhou, 399 Hailing Road, Taizhou, Jiangsu 225300, China; ^2^Department of Gastroenterology, Second Xiangya Hospital, Central South University, 139 Renmin Middle Road, Changsha, Hunan 410011, China

## Abstract

*Background*. MicroRNAs (miRNAs) have been found to be downregulated in human colorectal cancer (CRC), and some of them may function as tumor suppressor genes (TSGs). Aberrant methylation triggers the inactivation of TSGs during tumorigenesis.* Patients and Methods*. We investigated the methylation status of miR-125 family in CRC tissues and adjacent nontumor tissues by using bisulfite sequencing PCR (BSP). The expression levels of the two miRNAs were determined by quantitative reverse transcription polymerase chain reaction (qRT-PCR).* Results*. The methylation frequency of miR-125a and miR-125b was higher in CRC tissues. QRT-PCR analysis showed that miR-125a and miR-125b were significantly downregulated in CRC tissues. Moreover, the expression levels of miR-125a and miR-125b were inversely correlated to CpG island methylation in CRC.* Conclusions*. Our results suggest that DNA hypermethylation may be involved in the inactivation of miR-125a and miR-125b in CRC, and hypermethylation of miR-125 is a potential biomarker for clinical outcome.

## 1. Introduction

Colorectal cancer (CRC) is one of the most common malignancies and the fourth most frequent cause of cancer deaths worldwide. It is a heterogeneous disease, and both genetic and epigenetic events, such as genetic variations, DNA methylation, and histone modification, are involved in the initiation or development of CRC [1, 2]. The methylation of CpG dinucleotides in the promoter region of genes is an important mechanism of gene regulation, and aberrant DNA methylation patterns play a significant role in human tumor, including CRC [1, 2].

MicroRNA (miRNA) is a class of small noncoding RNAs, which regulate the expression of genes by inducing direct mRNA degradation or translational inhibition [3]. miRNAs can function as tumor suppressors or oncogenes in the regulation of biological processes, such as cell differentiation, proliferation, and apoptosis [4]. Accumulating evidence has shown that abnormal miRNA expressions contribute to human cancers [5, 6]. Although the biological importance of miRNA is becoming increasingly apparent, the molecular mechanisms underlying regulation of miRNA expression in cancer are not completely understood. Recent studies indicated that change in DNA methylation of promoter-associated CpG dinucleotides is an important mechanism leading to the dysregulation of miRNAs in cancers, including CRC [7]. Several tumor-associated miRNAs were found to be overexpressed or downregulated during CRC progression. Among these miRNAs, tumor-suppressive miRNAs, including miR-9, miR-129, miR-137, and miR-34a, are frequently silenced by aberrant DNA hypermethylation in CRC [7, 8].

The miR-125 family members have been reported to regulate tumor cell proliferation and metastasis. The family members are present in the mammalian genome: miR-125a localizes to chromosome 19q13, while miR-125b localizes to chromosome 11q23. Current studies revealed that the dysregulation of miR-125 occurs in multiple human cancer types [9–13]. High expression of miR-125 had been observed in gliomas and prostate cancer [14, 15], while miR-125 levels were demonstrated to decrease in breast and gastric cancer [11, 12]. To date, the expression level of miR-125 in CRC is not clear yet. The promoters of the two miR-125 family members are both embedded in CpG islands, according to the putative promoter regions. Considering the above findings, we wanted to test the expression and methylation status of miR-125 in CRC tissues and adjacent nontumor tissues and then to evaluate whether DNA methylation participates in regulating miR-125 expression in human CRC.

## 2. Materials and Methods

### 2.1. Clinical Tissue Samples

Specimens of CRC tissues and adjacent normal tissues from 68 CRC patients were obtained from Second Xiangya Hospital, Central South University, Hunan, China. Clinical data of the patients, including sex, age, tumor differentiation, lymph node metastasis, and clinical grade, was obtained from the hospital records. Patients who had undergone radiotherapy or chemotherapy prior to surgery were excluded from the study. Tumor samples were diagnosed in accordance with World Health Organization (WHO) system, by two pathologists who were unaware of patient data. The study was approved by the human ethics committee of Central South University. Informed consent was obtained from all subjects studied.

### 2.2. DNA Extraction and Bisulfite DNA

Genomic DNA was isolated from CRC tissues and adjacent nontumor tissues by using the Universal Genomic DNA Extraction Kit Ver. 3.0 (Takara, Dalian, Liaoning, China) in accordance with the manufacturer's instructions. The quality and integrity of DNA from these tissues were checked by electrophoresis on 1% agarose gel, quantified spectrophotometrically, and then stored at −20°C for further use.

### 2.3. Bisulfite Sequencing PCR

Bisulfite modification of DNA was produced by using the EpiTect Bisulfite Kit (Qiagen, Hilden, Germany) according to the manufacturer's recommendations. In brief, tissue DNA (1 *μ*g) was denatured using NaOH and subsequently modified by sodium bisulfite. Then, the mixture was desulfonated, and DNA was purified on silica-membrane columns to a final volume of 20 *μ*L. Bisulfite-modified DNA was stored at −20°C till further use. BSP was conducted as described previously [16]. The bisulfite-treated miR-125a promoter containing 17 CpG sites was amplified with the primers 5′-GATTTTAGGGTTTTTGATGAGG-3′ (sense) and 5′-ATCTCCTTAAATATCCTCCTCAACT-3′ (antisense), and the bisulfite-treated miR-125b promoter containing 20 CpG sites was amplified with the primers 5′-TTTGGGGAGGTTTTATATTTTT-3′ (sense) and 5′-AATAAAAAACATCTCTTTCCCC-3′ (antisense). The PCR products were subcloned into a pGEM T-Easy vector (Promega, Madison, WI, USA) after gel purification. The resulting products were next transformed into JM109 competent cells, and the blue/white screening was used to select a minimum of five bacterial transformants. Methylation states of each CpG site were analyzed by randomly sequencing 5–10 clones. The methylation level for each sample was calculated as the percentage of methylated CpG dinucleotides from the total number of CpG dinucleotides.

### 2.4. RNA Extraction and miRNA Detection

Total RNA was isolated from tissues with Trizol (Invitrogen, Carlsbad, CA, USA) following the manufacturer's instructions. RNA concentrations were determined with a NanoDrop instrument (NanoDrop Technologies, Wilmington, DE). Reverse transcription was performed using oligo dT primers or specific primers for miR-125 and control U6 (Ambion). Real-time PCR was carried out in an Applied Biosystems 7500 system using Power SYBR Green PCR master mix (Applied Biosystems). The real-time PCR experiments were repeated for three times.

### 2.5. Statistical Analysis

The difference of miR-125 promoter methylation status or expression levels between CRC tissues and adjacent normal tissues was examined by Student's *t*-test. The relationships of miR-125 methylation status and clinicopathological data were examined by the use of multivariate analysis. The expression levels of miR-125a and miR-125b between the hypermethylated group and methylated group were assessed using Mann-Whitney *U* test. Survival curves were estimated by the Kaplan-Meier method, and the differences in overall survival between subgroups were compared by the log-rank test. Results were regarded as significant when *P* was ≤0.05.

## 3. Results

### 3.1. miR-125 Expressions Are Downregulated in CRC

**Figure 1:**
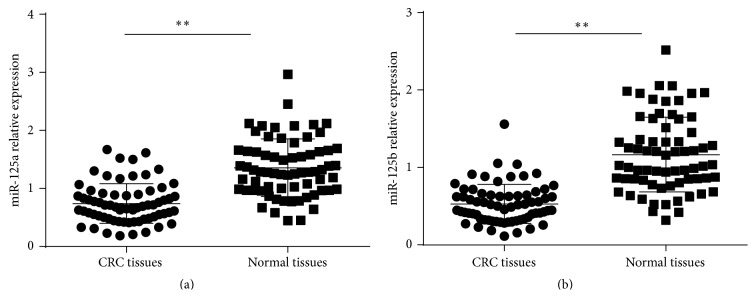
Difference between the expressions of miR-125 in 25 pairs of CRC tissues and adjacent normal tissues. The expression levels of miR-125a (a) and miR-125b (b) were significantly lower in tumor tissues compared with adjacent nontumor tissues. The expression levels of miRNAs were normalized to U6 expression and expressed as mean ± SD. ^*∗*^ *P* < 0.05.

Firstly, we measured the expression of miR-125 by using real-time PCR in 68 pairs of tumor and adjacent nontumor tissues. In Figures 1(a) and 1(b), the expression levels of miR-125a and miR-125b were significantly downregulated in CRC tissues compared with nontumor tissues. The relative expression levels of miR-125a <1.081 were considered as downregulation. The relative expression levels of miR-125b <0.79 were considered as downregulation.

### 3.2. miR-125a and miR-125b Are Hypermethylated in CRC

**Figure 2:**
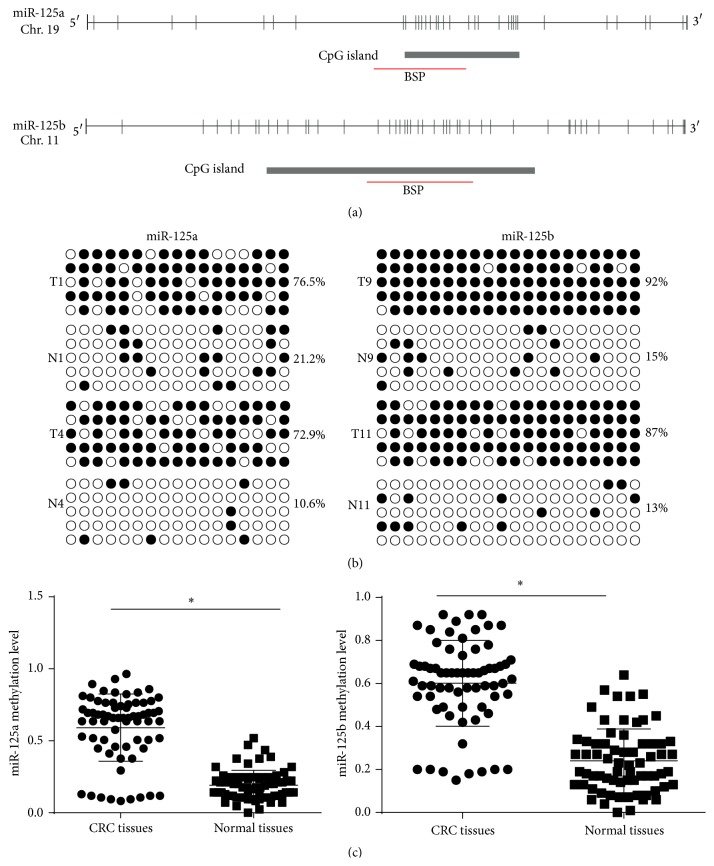
Bisulfite sequencing analysis of miR-125 in CRC and adjacent nontumor tissues. (a) Schematic diagrams show the distribution of CpG dinucleotides in the promoter region of miR-125a and miR-125b. (b) BSP of the upstream regulatory region of miR-125 was performed for representative tissues. For each sample, at least five separate clones were sequenced, and the results are shown here. Unmethylated CpG sites are shown as open circles, whereas methylated CpG sites are indicated by closed circles. For each row of circles, the sequence results for an individual clone of the bisulfite-PCR product are given. The number of methylated CpGs divided by the total number of true CpGs analyzed is given as a percentage on the right of each BSP result. (c) The methylated levels of miR-125a and miR-125b were significantly higher in tumor tissues compared with adjacent nontumor tissues. The results were expressed as mean ± SD. ^*∗*^ *P* < 0.05.

To investigate whether epigenetic alteration could be extrapolated to CRC, we performed BSP analysis to determine the methylation status in the promoter regions of miR-125 family in 68 pairs of CRC tissues and adjacent nontumor tissues. We designed and validated bisulfate sequencing PCR for the promoter region of miR-125a including 17 CpGs and that of miR-125b including 20 CpGs, respectively (Figure 2(a)). The results showed that the CpG sites were highly methylated in tumor tissues for miR-125a, and the methylation level varied from 8.2% to 96.5%, with a mean ratio of 59.12% in the tumor tissue (Figures 2(b) and 2(c)). In contrast, the methylation level of miR-125a observed in the adjacent nontumor samples ranged from 0% to 51.8%, with a mean of 19.17% (Figures 2(b) and 2(c)). Methylation levels >35.77% were considered as hypermethylation. miR-125a was hypermethylated in 58 (85.3%) of 68 tumors. For miR-125b, the methylation level varied from 15% to 92%, with a mean ratio of 60.12% in the tumor tissue (Figures 2(b) and 2(c)), whereas the methylation level of miR-125b observed in the adjacent nontumor samples ranged from 0% to 64%, with a mean of 20.20% (Figures 2(b) and 2(c)). Methylation levels >40.2% were considered as hypermethylation. miR-125b was hypermethylated in 59 (86.7%) of 68 tumors. We then evaluated the association of methylation status of miR-125a and miR-125b in patients with different clinicopathologic characteristics including sex, age, histological differentiation, lymph node metastasis, and grade. However, no significant difference was found between the subgroups of patients (Tables 1 and 2). And each miRNA is an independent parameter for the poor prognosis.

### 3.3. Associations between miRNAs Methylation and miRNAs Expression

**Figure 3:**
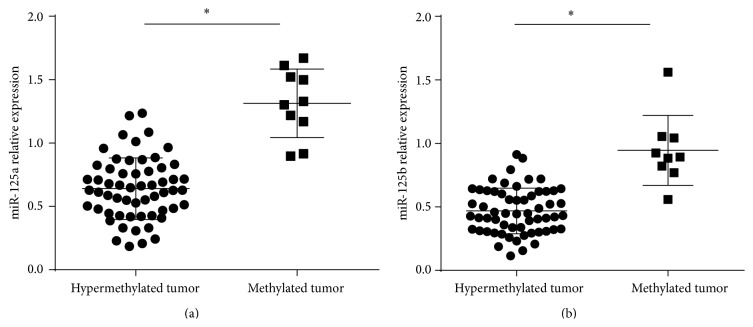
Associations between promoter methylation and miR-125 family expression among 68 CRC tissues. The expression levels of miR-125a (a) and miR-125b (b) were compared between hypermethylated group and methylated group in tumor tissues. The results showed that the expression levels of miR-125a and miR-125b were significantly lower in hypermethylated tumor group than in methylated tumor group. ^*∗*^ *P* < 0.05.

Aberrant promoter methylation is considered a hallmark of cancer involved in silencing of tumor suppressor genes and activation of oncogenes. Aberrant DNA methylation includes hyper/hypomethylation. For tumor suppressor genes, the molecules are commonly hypermethylated in tumor tissues compared with nontumor tissues, while oncogenes are often hypomethylated. In our study, we defined cases with aberrant methylation as hypermethylated tumors and cases without aberrant methylation as methylated tumor tissues. To further determine whether DNA methylation contributes to the silencing of miR-125 family in CRC, we compared the differences of miR-125a and miR-125b between hypermethylated and methylated groups in tumor tissues (Figure 3). As shown in Figures 3(a) and 3(b), the expression levels of miR-125a and miR-125b were significantly lower in the hypermethylated tumor group than that in the methylated tumor group.

### 3.4. Clinical Outcome of the Patients

**Figure 4:**
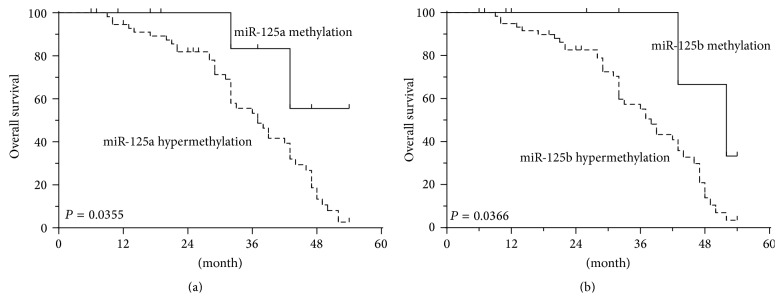
Kaplan-Meier analysis of overall survival in relation to miR-125a and miR-125b methylation status. (a) The correlation between miR-125a methylation in the tumor tissue and the OS of the CRC patients. The patients with hypermethylation of miR-125a had a shorter OS than those with normal levels. (b) The correlation between miR-125b methylation in the tumor tissue and the OS of the CRC patients. The patients with hypermethylation of miR-125b had a shorter OS than those with normal levels.

To determine the prognostic value of miR-125 members for CRC, we analyzed the association between methylation status of miR-125a or miR-125b and survival duration by using the Kaplan-Meier analysis. The results showed that CRC patients with hypermethylated promoter of miR-125a or miR-125b in tumors revealed shorter overall survival (OS) as compared to patients without hypermethylated promoter (*P* = 0.0355, *P* = 0.0366, Figures 4(a) and 4(b)).

## 4. Discussion

Colorectal carcinogenesis is a multistep process involving the dysregulation of oncogenes and tumor suppressor genes. Recent studies have shown that microRNA-associated transcriptionally regulated gene expression plays a critical role in the initiation and development of CRC, through regulating cell proliferation, cell cycle, apoptosis, and metastasis. The miR-125 family has been reported to be involved in multiple cancers. Depending on cell context, miR-125a and miR-125b function as oncogenes or tumor suppressors. miR-125a was significantly downregulated in breast and gastric cancer, and miR-125a substantially inhibited the proliferation, migration, and invasion activities of cancer cells [11, 12]. Clinical samples were found to overexpress miR-125b in prostate cancer, and enforced expression of miR-125b promoted tumor growth through mediation of broad attenuation of the intrinsic apoptosis pathway by targeting P53, PUMA, and BAK [17]. miR-125b has also been observed to be upregulated in gliomas, and it promotes proliferation and inhibits apoptosis in gliomas cells [14]. In contrast, in breast cancer, miR-125b levels were demonstrated to decrease compared with normal nontumor tissue. Furthermore, miR-125b was found to suppress the oncoproteins MUC1, ERBB2, and ERBB3, inhibiting the growth of breast cancer cells [12]. However, the role of miR-125 in CRC is not clear yet. In the present study, we showed that miR-125a and miR-125b were frequently downregulated in CRC tissues, which suggested the tumor-suppressive properties of the miR-125 family in CRC.

Aberrant DNA methylation has critical effects on CRC initiation and progression, and both DNA hypermethylation of specific genes and miRNAs are observed in CRC. Human miR-125a is located at 19q13, while miR-125b is located at 11q23. The promoters of the two miRNAs were both located within CpG islands. The expression levels of the two miRNAs may be both regulated by DNA methylation. Zhang et al. observed DNA hypermethylation of miR-125b in human breast cancer [18]. Kim et al. also demonstrated the restoration of miR-125a and miR-125b expression in hepatocellular carcinoma following 5-Aza-dC treatment [19]. In this investigation, we computationally mapped CpG-rich regions in the promoter of miR-125a and miR-125b. Hypermethylation of miR-125a and miR-125b promoter was found in CRC, and further analysis showed that the expressions of the two miRNAs were significantly lower in hypermethylated tumor tissues than that in methylated counterparts, suggesting that aberrant CpG methylation in the promoter region of miR-125a and miR-125b might silence its expression in CRC tumorigenesis. Kaplan-Meier analysis showed that miR-125 methylation status was inversely correlated with overall survival of the CRC patients. These findings indicated that hypermethylation of miR-125 family member may be involved in the tumorigenesis of CRC, and it could be a good biomarker for the clinical outcome in CRC patients.

In conclusion, we report that miR-125a and miR-125b expression is downregulated in CRC tissues and hypermethylation of miR-125a and miR-125b promoter partially accounts for the reduction of the two miRNAs' expression. Further studies in vitro and in vivo are required to validate the value of miR-125 in CRC tumorigenesis.

## Figures and Tables

**Table 1:** Clinicopathological features of CRC patients and BSP analysis of miR-125a in tumor tissues.

Variable	Hypermethylation and low expression	Hypermethylation and high expression	Methylation and low expression	Methylation and high expression	*P* value
Sex					0.686
Male (46)	38	2	1	5	
Female (22)	17	1	1	3	
Age (years)					0.343
≤50 (16)	13	1	0	2	
>50 (52)	42	2	2	6	
Histological differentiation					1.000
Good or moderate	31	1	1	6	
Poor or none	24	2	1	2	
Lymph node metastasis					0.686
Negative	28	1	1	8	
Positive	24	2	1	3	
Grade					0.886
Low grade (I + II)	25	1	1	8	
High grade (III + IV)	30	2	1	0	

**Table 2:** Clinicopathological features of CRC patients and BSP analysis of miR-125b in tumor tissues.

Variable	Hypermethylation and low expression	Hypermethylation and high expression	Methylation and low expression	Methylation and high expression	*P* value
Sex					0.486
Male (46)	38	2	2	4	
Female (22)	18	1	0	3	
Age (years)					1.000
≤50 (16)	12	1	1	2	
>50 (52)	45	1	1	5	
Histological differentiation					0.486
Good or moderate	31	3	1	4	
Poor or none	25	0	1	3	
Lymph node metastasis					1.000
Negative	32	1	1	4	
Positive	22	2	1	3	
Grade					0.686
Low grade (I + II)	31	0	1	3	
High grade (III + IV)	26	2	1	4	
